# Surgical and clinical impacts of mixed reality-guided glioblastoma resection versus standard neuronavigation: improving tumor surgery

**DOI:** 10.3389/fonc.2025.1551937

**Published:** 2025-03-21

**Authors:** Julien Haemmerli, Samuel Khatchatourov, Etienne Chaboudez, Leonard Roth, Abiram Sandralegar, Insa Janssen, Denis Migliorini, Karl Schaller, Philippe Bijlenga

**Affiliations:** ^1^ Division of Neurosurgery, Department of Clinical Neurosciences, Geneva University Hospitals, Geneva, Switzerland; ^2^ Faculty of Medicine, University of Geneva, Geneva, Switzerland; ^3^ Centre for Primary Care and Public Health (Unisanté), University of Lausanne, Lausanne, Switzerland; ^4^ Department of Oncology, Geneva University Hospitals, Geneva, Switzerland

**Keywords:** mixed reality, oncologic neurosurgery, glioblastomas, GTR, progression-free survival

## Abstract

**Background:**

Glioblastomas (GBM) are typically treated with surgery and radio-chemotherapy, with patient survival often depending on the extent of tumor resection. This study compares outcomes of GBM surgery using 5-ALA, intraoperative neuroelectrophysiology, and neuro-navigation, either in a standard setting (STD) or enhanced by mixed reality (MR) guidance.

**Methods:**

This retrospective study included GBM patients who underwent resection at Geneva University Hospitals between 2015 and mid-2022, excluding biopsies and partial debulking. Primary outcomes included postoperative residual tumor volume (RV) based on postoperative contrast uptake on the MRI, while secondary outcomes were gross total resection (GTR), extent of resection (EOR), new postoperative deficits, overall survival (OS), progression-free survival (PFS), and Karnofsky performance scores. Confounding factors such as intraoperative monitoring and use of fluorescence were analyzed.

**Results:**

Of 115 patients, 76 were in the STD group and 39 in the MR group, with comparable demographics. The MR group had significantly lower RV (median 0.01 cm³ vs. 0.34 cm³, p=0.008) and higher GTR rates (median 50% vs. 26.7%). EOR was also superior in the MR group (median 99.9% vs. 98.2%, p=0.002). New focal deficits occurred in 39% (STD) and 36% (MR) of cases (p=0.84). While median OS was not significantly different (475 vs. 375 days, p=0.63), median PFS was longer in the MR group (147 vs. 100 days, p=0.004).

**Conclusion:**

MR guidance improves the quality of tumor resection and enhances progression-free survival without increasing postoperative deficits, although it does not significantly impact overall survival.

## Highlights

- Surgically, mixed reality improves the quality of resection in glioblastomas.
- Patients operated under mixed reality have longer progression-free survival.
- No difference regarding functional status was found between MR and navigation group.

## Introduction

Glioblastoma (GBM) is the most frequent and aggressive primary brain tumor, with a median overall survival (OS) reported between 15 to 16 months with optimal treatment (1, 2). The standard treatment strategy involves surgical resection of the contrast-enhanced volumes on the preoperative MRI, followed by radio-chemotherapy plus high doses of temozolomide (3, 4). Numerous studies have demonstrated that both the quality of resection and postoperative clinical outcomes significantly influence median survival rates (5–8). Although survival prognosis is also affected by preoperative factors such as age, performance status, tumor volume, location, and molecular markers (9, 10), achieving maximal extent of resection (EOR) and minimizing residual tumoral volume (RV) without inducing new neurological deficits are critical surgical priorities. Progression-free survival (PFS), which correlates with overall survival (11), is similarly dependent on GTR, EOR and RV (12).

To enhance surgical precision, various intraoperative techniques have been developed over the past two decades, including fluorescence guidance using 5-aminolevulinic acid (5-ALA) and sodium fluorescein (13, 14), as well as neuronavigation systems (15).

Recently, augmented reality (AR) and mixed reality (MR) technologies have garnered interest in neurosurgery as advanced navigation systems (16, 17). AR involves overlaying virtual images onto the real world, whereas MR encompasses various interactions between virtual and real structures (18). In neurosurgery, MR provides an interactive intraoperative 3D view, enabling the surgeon to project preoperatively defined virtual structures, such as white fiber tracts, vessels, or tumor contours, onto the surgical field. These technologies aims to improve navigation precision (17). However, only a few studies have investigated the surgical or clinical impact of MR on cranial tumor resection (16, 19, 20), and none have focused exclusively on GBM while isolating MR-effect.

This retrospective study aims to compare the surgical and clinical outcomes of GBM patients who underwent surgery using either standard neuronavigation (STD) or MR, while controlling for other confounding surgical factors.

## Materials and methods

### Population

This retrospective study analyzed patients with GBM who underwent primary surgery at the Geneva University Hospitals, from January 1^st^ 2015 to June 30^th^, 2022, with a follow-up until July 1^st^, 2023. Patients were included based on the following criteria: 1) adults, 2) histopathological and molecular diagnosis fitting the criteria of GBM (21), 3) one cerebral MRI performed within days before the surgery and one within 48 hours post-surgery, 4) surgical intent to completely resect contrast uptake on T1 + gadolinium sequence for unilocular lesions, or 5) intent to completely resect at least one contrast uptake location for multilocular contrast uptake.

Excluding criteria were: 1) previous therapeutic interventions such as surgery, radiotherapy, or chemotherapy, 2) planned biopsy or partial debulking, 3) emergency situations precluding the use of mixed reality (MR), 4) age under 18, 5) histopathological diagnosis not meeting the GBM criteria.

Eligible patients were screened from the local neuro-oncology tumor board database.

### Ethics statement

All included patients were informed about the navigation technique used for surgery and signed surgical consent forms. Both STD and MR are established and recognized navigation techniques for tumor resection. This project follows the principles outlined in the *World Medical Association Declaration of Helsinki: Research involving human subjects*.

### Data extraction

Volumetric analyses were assessed using the Element^®^ software (Brainlab, Munich, Germany). Preoperative tumoral volumes and postoperative tumor residual volumes (RV) were manually contoured using the MRI subtraction sequence, or T1 gadolinium-enhanced MP-RAGE sequence minus superimposed on a native T1 sequence if subtraction sequences were unavailable. Analyzed volumes were automatically calculated and expressed in cm^3^. The volumetry was performed independently by two examiners (SK and EC). An inter-rater reliability was assessed using the intraclass correlation coefficient (ICC,3k).

For multilocular lesions, defined as two or more areas with distinct contrast enhancement on the most recent preoperative MRI, only the volume of the lesions preoperatively intended to be resected was considered for the volumetric analyses. In cases where multiple contrast uptake locations were intended to be removed, the preoperative tumor volume was calculated by summing the volumes of the different contrast-enhanced areas.

To assess the postoperative cavity volume dimension relative to the preoperative tumoral volume, a volume difference (Δvol) was calculated by subtracting the preoperative tumoral volume from the sum of the cavity volume and the RV.

Lesions were characterized as eloquent if they were located less than 1 cm away from the following areas: primary motor cortex, primary somatosensory cortex, primary visual cortex, Broca’s area, Wernicke’s area, thalamus, and basal ganglia.

In certain instances, alarms from intraoperative neuromonitoring (IONM) or surgical decision to limit the resection for safety purposes restricted the surgeon’s ability to achieve complete macroscopic resection (CMR). Therefore, the post-operative residual volume was subcategorized based on the surgeon’s evaluation at the end of the procedure of having achieved CMR or not, as documented in the operative reports.

Clinical data were collected through a thorough examination of the patients’ medical records, and direct contact with their physicians or oncologists when follow-up information was missing. All patients followed a standardized post-surgical protocol, including admission to intensive or intermediate care units and continuous care units after surgery. A systematic post-operative MRI was performed within 48 hours after surgery. Each operated case was discussed in a multidisciplinary tumor board panel, which provided the preferred adjuvant therapy schema. The standard adjuvant treatment (STUPP schema (3, 4)) or less aggressive options were decided on a case-specific basis, considering the patient’s clinical condition, surgical outcomes, and prognosis. After surgery, patients were either discharged or sent to rehabilitation. Patients were systematically called in Neurosurgery for a follow-up between six weeks and three months, and regular contact with oncology and radiology specialists constituted the basis of the follow-up. Control MRI schedules were individualized, typically every three months initially, with adjustments based on radiological and clinical assessments.

When Karnofsky performance scale (KPS) scores were not recorded, they were estimated from clinical data and other documented scales [e.g. Eastern Cooperative Oncology Group (ECOG) scale (22)], to ensure a systematic assessment of patient status. If the available data was too limited for reliable estimates, KPS scores were excluded from the analysis.

New postoperative focal neurological deficits were defined as any new focal sign or symptom observed within the first two weeks after the surgery and not restored at six weeks or during rehabilitation. A documented worsening of a preoperative focal deficit was also classified as a new postoperative deficit. Spatial neglect was systematically considered as a focal deficit.

Surgical complications were defined as adverse events occurring during surgery, or any surgery-related event that prolonged the patient’s hospital stay, necessitated readmission, or required additional interventions.

PFS was determined on the basis of the existing radiological reports, evaluated in a single-blind manner, with the evaluators unaware of whether patients had received MR-based surgery or not. Any increase in contrast enhancement, including the enlargement of a residue or a satellite lesion that was not targeted by surgery, suggestive of high-grade progression and not visible on comparative imaging, was classified as a progression.

### Data availability statement

Authors agree to make data and materials supporting the results of this study in an anonymous fashion available upon reasonable request. It is up to the authors to consider whether a request is reasonable or not. Any request should be addressed directly to the corresponding authors (JH).

### Definition of the compared groups

Patients in this cohort underwent surgery using either mixed reality (MR group) or standard neuronavigation (STD group). There was no randomization; the choice of navigation method was based on the surgeon’s preferences. In both groups, involved surgeons were comparable in terms of experience regarding GBM resection. The neurosurgeons participating in the MR and STD groups were the same. It is worth noting that authors JH and PB have the most extensive experience with MR and utilized this technique more frequently than the other participating staff neurosurgeons for GBM removal. For the purpose of this study, patients were retrospectively assigned to one of the two groups according to the neuronavigation methods used by the neurosurgeon (mixed reality versus standard neuronavigation). All involved surgeons were board-certified in Neurosurgery and were trained for GBM resection.

### Neuronavigation processes

All patients enrolled in the study benefited from a preoperative MRI as part of the routine diagnostic workup. The images were loaded onto the Element^®^ software (Brainlab, Munich, Germany).

#### Mixed reality

**Figure 1:**
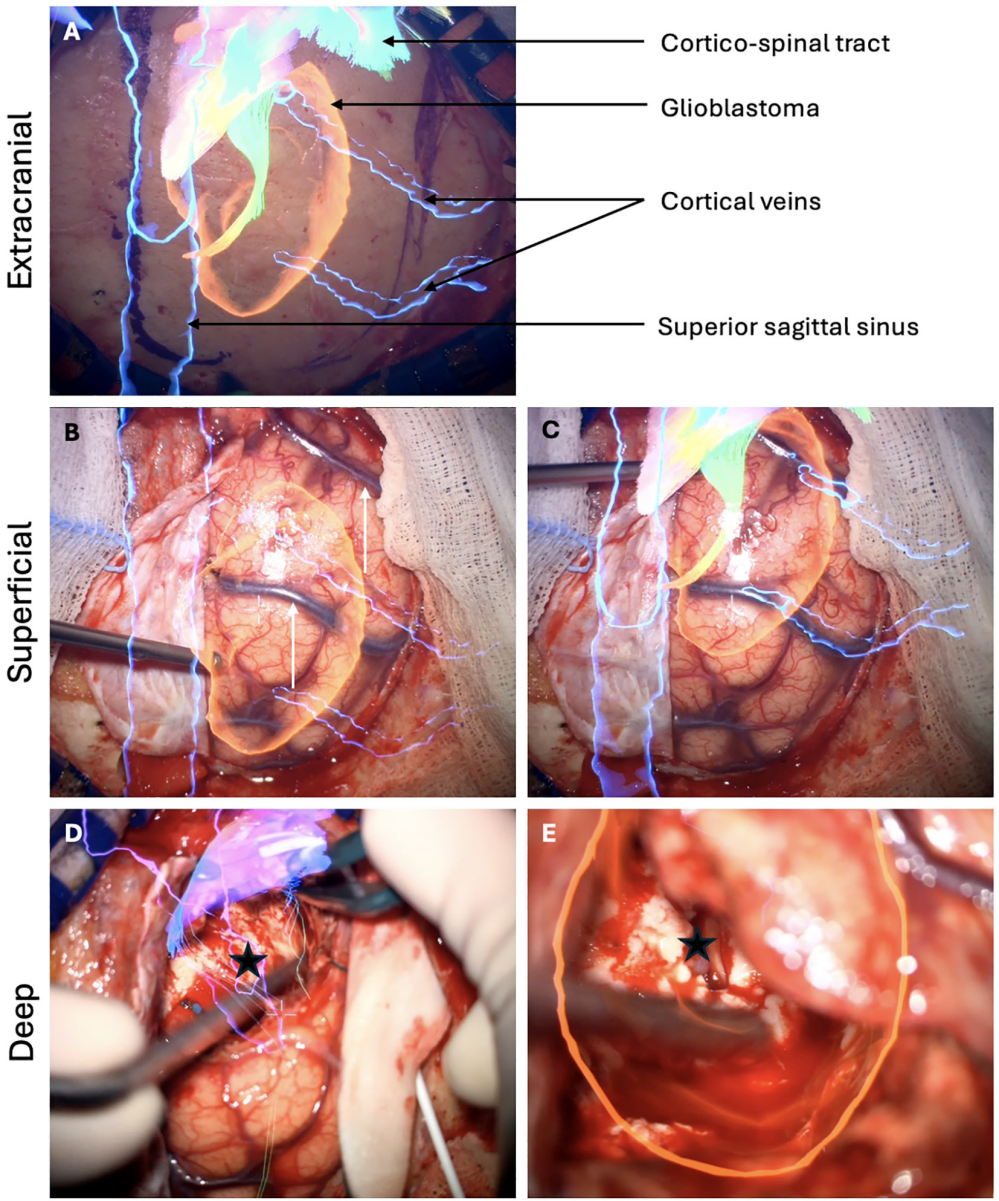
Sequential steps of MR recalibration during surgery for a right parietal parasagittal glioblastoma extending until the right lateral ventricle. **(A)** Following the skin incision, MR is checked according to the bone fissures of the skull. The overlay of the superior sagittal sinus indicates the midline. The planned craniotomy is guided by the tumor's location and the position of the corticospinal tract. **(B)** After dura opening, a shift is observed in the alignment of the two cortical veins used as reference structures. **(C)** MR recalibration restores optimal alignment between the virtual and real images of the cortical veins. **(D)** As the resection advances, MR indicates that the roof of the right lateral ventricle should be reached at this stage (black star). **(E)** Inspection of the surgical cavity confirms the opening of the lateral ventricle roof (black star), verifying the navigation system's accuracy and confirming the completeness of the deep resection.

In the Element^®^ software (Brainlab, Munich, Germany), automatic segmentation of standard structures such as brain, skull, optic nerves, ventricles, or brainstem was available. Tumor borders were manually designed, in addition to neighboring structures of interest and other elements regarding the surgical roadmaps (16). In case involving eloquent region, specific white matter tracts - such as the cortico-spinal tract, optic tract, superior lateral frontal tracts I-II-II or inferior lateral tract - were delineated using the Element^®^ software. Each tract defined as an individual object and could be displayed through the surgical microscope either separately or in combination. Intraoperatively, the surgical microscope was initially registered with the navigation system. To ensure continuous optimal accuracy, MR was manually recalibrated on the navigation workstation using the surgical microscope in a sequential out-to-in fashion based on signature structures. Before the skin incision, accuracy was verified using the patient’s virtual 3D facial model, focusing on key landmarks such as the internal epicanthi and nasion (x- and y-axes) and the external auditory canal (y-axis). After the skin incision, accuracy was reassessed by verifying the alignment of virtual and real images of sutures or bone landmarks. Following dura opening, meticulous attention was paid to the correlation between superficial vessels and their corresponding virtual 3D models or the projection of the MRI on the surgical field. While the resection was progressing, MR recalibration was performed according to deeper structures such as vessels or ventricles. As the resection progressed, MR recalibration was carried out based on deeper structures, such as vessels or ventricles (**Figure 1**). This approach compensates for any brain shift caused by sagging or displacement of anatomical structures during the procedure, thereby minimizing MR inaccuracies. Finally, tumor boundaries were verified using both MR projections and 5-ALA fluorescence.

#### Standard neuronavigation

In cases using standard neuronavigation (Brainlab, Munich, Germany) was employed. Registration was performed using a navigated pointer and was based on patients’ face landmarks, including eyebrows, epicantii, nose, front. The procedure has been previously described in the literature (23, 24). Unlike MR, standard neuronavigation does not allow for intraoperative readjustments or 3D rendering. Only axial, coronal, and sagittal views of the preoperative MRI were available to the operator.

#### Confounding factors

The use of IONM as case-specific and implemented upon the surgeon’s request based on lesion location in both groups. Similarly, intraoperative ultrasonography and intraoperative MRI (the latter introduced in 2021) were employed on a case-by-case basis. For fluorescence guidance, all patients were administered 5-ALA prior to surgery.

### End points

#### The primary outcome was the residual tumor volume

The secondary outcomes included: gross total resection (GTR, defined as the complete resection of the preoperative contrast uptake on the preoperative MRI), extent of resection (EOR, defined as the percentage of resected tumor), duration of surgery, intra- and postoperative complications, Karnofsky performance scale scores (KPS) at 6 weeks and 3 months, incidence of new post-operative focal neurological defects, progression-free survival (PFS) and overall survival (OS).

### Statistical analysis

The statistical analysis was performed using the R software environment, version 4.3.1, and RStudio, version 2023.06.1 + 524. The normality of each distribution was assessed using a Shapiro test. Depending on the distribution assessment, medians or means were compared using Mann-Whitney U tests or Student’s t tests, respectively. Proportions were compared using Fisher’s exact tests. The intraclass correlation coefficient (ICC) was calculated to evaluate the agreement between the two raters (SK and EC) (two-way random effects, consistency, multiple raters, ICC (3, *k*)). An ICC<0.5 was considered as poor, ≥0.5 and <0.75 as moderate, ≥ 0.75 and <0.9 as good, and ≥ 0.9 as excellent agreement (25). The ICC (3,2) for preoperative tumor volumetry was excellent (0.97 (95%-CI 0,96-0,98, p<0.001)) as well as for the postoperative tumor measurement (0,99 (95%-CI 0.98 – 0.99, p<0.001)).

Survival analyses were performed by first deriving the Kaplan-Meier estimator and testing differences between survival curves with the log-rank test. Then, Cox proportional hazard models were built in two steps to adjust for confounding prognostic factors and give appropriate hazard ratios for MR. First, all factors potentially impacting the neuronavigation process based on the investigators’ knowledge as well as those established in the literature as partly determining survival were considered (26, 27). Second, the variables that were associated neither with the neuronavigation nor with the survival outcomes were discarded in the final models. This step was necessary due to the noncollapsbility of the Cox model, which may induce bias in the isolated effect of MR. To stay conservative, a p-value cut-off of 0.1 was chosen in this case. In all other cases, a p-value lesser than 0.05 was considered significant.

## Results

### Perioperative groups comparison

**Table 1** presents the demographic and tumor characteristics in the groups. The STD group included 76 patients, and the MR group 39, totalizing 115 patients presenting a newly diagnosed GBM. Demographic characteristics were comparable between the STD and MR groups, with respective mean ages of 64.2 (SD 10.4) and 61.5 (SD 11.1) and female ratios of 37% and 49%. Preoperative KPS score was similar between the groups, with a median of 90/100 (Q1 = 80, Q3 = 90, p=0.83). Preoperative focal neurological defects concerned 76% of patients in the STD group and 64% in the MR group (p=0.19).

**Table 1:** Preoperative and intraoperative data.

	STD group (n = 76)	MR group (n = 39)	Missing values (n)	p-value
Group data				
Age (mean, (sd))	64.2 (10.4)	61.5 (11.1)	0	0.21
Sex (female ratio)	37 %	49 %	0	0.24
Preoperative KPS (med, (Q1, Q3)) *	90 (80, 90)	90 (80, 90)	30	0.83
Preoperative focal deficit	76 %	64 %	0	0.19
Lesions description				
Multiple lesions **	34 %	18 %	0	0.08
Intention to remove all lesions	82 %	95 %	0	0.08
Side (right)	61 %	54 %	0	0.16
Eloquent area involvement	46 %	49 %	0	0.84
Targeted lesion(s) size [cm^3^] (med (Q1, Q3)) ***	27.9 (12.10, 45.15)	27.10 (12.7, 45.95)	1	0.90
Localization			0	0.17
*Frontal involvement*	42 %	36 %		
*Parietal involvement*	28 %	46 %		
*Temporal involvement*	42 %	41 %		
*Occipital involvement*	12 %	10 %		
*Central (corpus callosum)*	0%	3 %		
*Ventricular origin*	0%	3 %		
*Exclusively frontal*	36 %	28 %	0	0.53
*Exclusively temporal*	32 %	18 %	0	0.18
Surgical methods				
5-ALA	100 %	100 %	0	1
IONM	61 %	80 %	0	0.06
Intraoperative MRI	4 %	8 %	0	0.41
Intraoperative US	11 %	3 %	0	0.16
Surgical time				
Minutes (med (Q1, Q3))	238 (192, 288)	270 (228, 322)	0	0.005
Genetics				
MGMT methylation	41 %	28 %	0	0.22

* The preoperative KPS score of 30 patients (25 from the STD group, 5 from the MR group) was not documented and could not be reconstructed with sufficient accuracy

** Multiple lesions are defined as at least to separate lesions on the preoperative MRI

*** Size of the lesion(s) preoperatively planned to be removed by surgery. The one missing data is due to the loss of the preoperative MRI.

KPS, karnofksy performance scale; MGMT, O-6-methylguanine-DNA methyltransferase; 5-ALA, δ-Aminolevulinic acid; IONM, intra-operative neuro-monitoring; STD, standard group; MR, mixed reality group.

Targeted lesion volumes were also comparable, with a median of 27.9 cm^3^ and 27.1 cm^3^ for the STD and MR groups, respectively (p=0.9). Tumor locations did not significantly differ. Eloquent area implication was as in the STD as in the MR group (respectively 46% and 49%). 34% and 18% of lesions were multifocal in the STD and MR groups (p=0.08), respectively, and the intention to remove all lesions was of 82% and 95% (p=0.08).

Regarding the intraoperative methods helping for tumor detection and patients’ safety, the use of 5-ALA was systematic in all included patients, whereas IONM was employed in 61% of the STD and 80% of the MR cases (p=0.06). Additional intraoperative specific imagery did not significantly differ between the two groups, as intra-operative MRIs were realized in 4% and 8% of the cases of the STD and MR groups (p=0.41), respectively, and intra-operative ultrasound was used 11% and 3% of the time (p=0.16).

Regarding the histopathological results, a MGMT-promoter methylation was found in 41% of the STD and 28% of the MR cases (p=0.22).

The operation time was significantly shorter in the STD group (median times: STD = 238 min, MR = 270 min, p=0.005).

### Tumor resection

**Figure 2:**
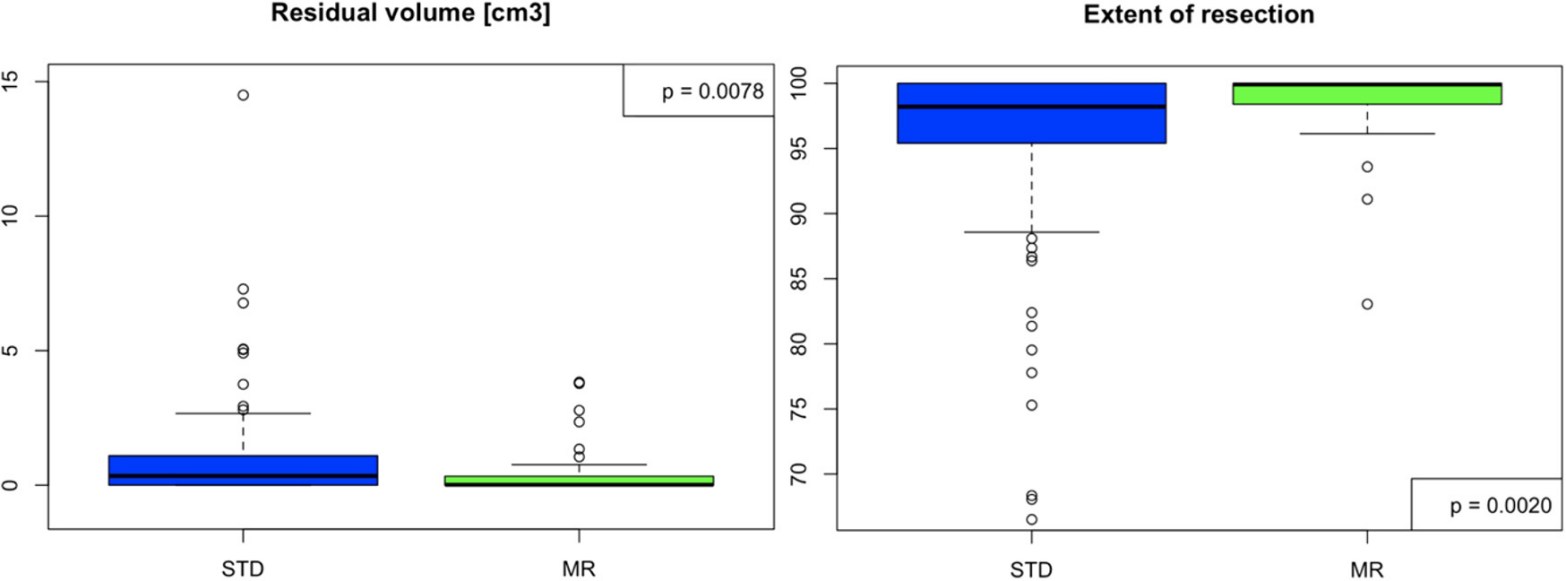
Results of the postoperative volumetry between STD and MR group. STD, Standard navigation group; MR, Mixed reality group.

**Table 2** shows the surgical outcomes between groups. In 87% of cases across both groups, surgeons assessed they had achieved complete macroscopic resection (CMR) at the end of the procedure. Overall, the RV was significantly smaller in the MR group, which had a median volume of 0.01 [cm^3^] (Q1 = 0, Q3 = 0.33), compared to the STD group, with a median volume of 0.34 (Q1 = 0, Q3 = 1.09, p=0.008) (**Figure 2**). Accordingly, GTR was greater in the MR group (50%) in comparison to the STD group (26.7%) (p=0.021). Also, the EOR was significantly higher in the MR group (median=99.91, Q1 = 98.49, Q3 = 100) than in the STD group (median=98.21, Q1 = 95.40, Q3 = 100, p=0.002) (**Figure 2**).

**Table 2:** Results of the volumetry per group.

	Median	Q1	Q3	Missing values (n)*	p-value
**RV total [cm^3^]**					0.008
STD (n=76)	0.34	0.00	1.09	1	
MR (n=39)	0.01	0.00	0.33	1	
**GTR [%]**					0.021
STD (n=76)	26.7			1	
MR (n=39)	50.0			1	
**EOR total [%]**					0.002
STD (n=76)	98.21	95.40	100.00	1	
MR (n=39)	99.91	98.49	100.00	1	
**RV in case of CMR [cm^3^]**					0.009
STD (n=66)	0.21	0.00	0.86	1	
MR (n=35)	0.00	0.00	0.30	0	
**EOR in case of CMR [%]**					0.003
STD (n=66)	98.32	96.51	100.00	2	
MR (n=35)	100.00	99.20	100.00	0	
**Δvol**					0.76
STD (n=76)	- 0.02	- 8.95	3.64	2	
MR (n=39)	- 4.45	- 11.17	4.28	1	

* The missing values are due to lack of contrast-enhancing sequences in the postoperative MRIs, the loss of one preoperative MRI and the inability in one case to distinguish between a deliberately left or unintentionally missed residual contrast enhancement on a postoperative MRI.

RV, residual volume; GTR, gross total resection; EOR, extent of resection; CMR, complete macroscopic resection; ∆vol, cavity volume + RV volume – preoperative tumoral volume; STD, standard group; MR, mixed reality group.

When focusing on the subgroup of patients with CMR, the median RV in the STD group was 0.21 (Q1 = 0, Q3 = 0.86), and 0 (Q1 = 0, Q3 = 0.30) in the MR group (p=0.009). The EOR of the STD group was lower than in the MR group, with respective medians of 98.32 and 100 (p=0.003). The volume difference (Δvol) was similar across both groups.

### Clinical outcomes

**Table 3** summarizes the clinical outcomes between groups. The overall complication rate was of 21% and 13% (p=0.32) for the STD and MR groups, respectively. No complication was specifically attributable to the use of MR or standard neuronavigation. The rate of new postoperative focal deficits was 36% and 39% (p=0.84). The KPS scores at 6 weeks were similar (for both navigation methods, median=80, Q1 = 70, Q3 = 90, p=0.38) as well as at 3 months (median=80, Q1 = 70, Q3 = 90, p=0.66). The difference with the pre-operative KPS score was also comparable between the groups, with a median of 0 at 6 weeks (p=0.58) and at 3 months (p=0.94).

**Table 3:** Results regarding operative and clinical outcomes.

	STD group (n = 76)	MR group (n = 39)	Missing values (n)	p-value
Peri- and post-procedural outcomes				
Operation time [min] (med (Q1, Q3))	238 (192, 288)	270 (228, 322)	0	0.005
Complications*	21 %	13 %	2	0.32
Clinical outcomes				
New postoperative deficit	36 %	39 %	0	0.84
KPS at 6 weeks (med (Q1, Q3)) **	80 (70, 90)	80 (70, 90)	15	0.38
ΔKPS at 6 weeks (med (Q1, Q3)) ***	0 (-10, 0)	0 (-10, 0)	38	0.58
KPS at 3 months (med (Q1, Q3)) **	80 (70, 90)	80 (70, 90)	16	0.66
ΔKPS at 3 months (med (Q1, Q3)) ***	0 (-10, 0)	0 (-10, 0)	38	0.94

* Follow-up for 2 patients were incomplete due to transfer in other countries, one from each group.

** The 6-week postoperative KPS score of 15 patients (13 from the STD group, 2 from the MR group) was not documented and could not be reconstructed with sufficient accuracy, as well as the 3-months score of 16 patients (15 from the STD group, 1 from the MR group).

*** The Δ is calculated by subtracting the preoperative KPS score to the postoperative KPS score.

**Figure 3:**
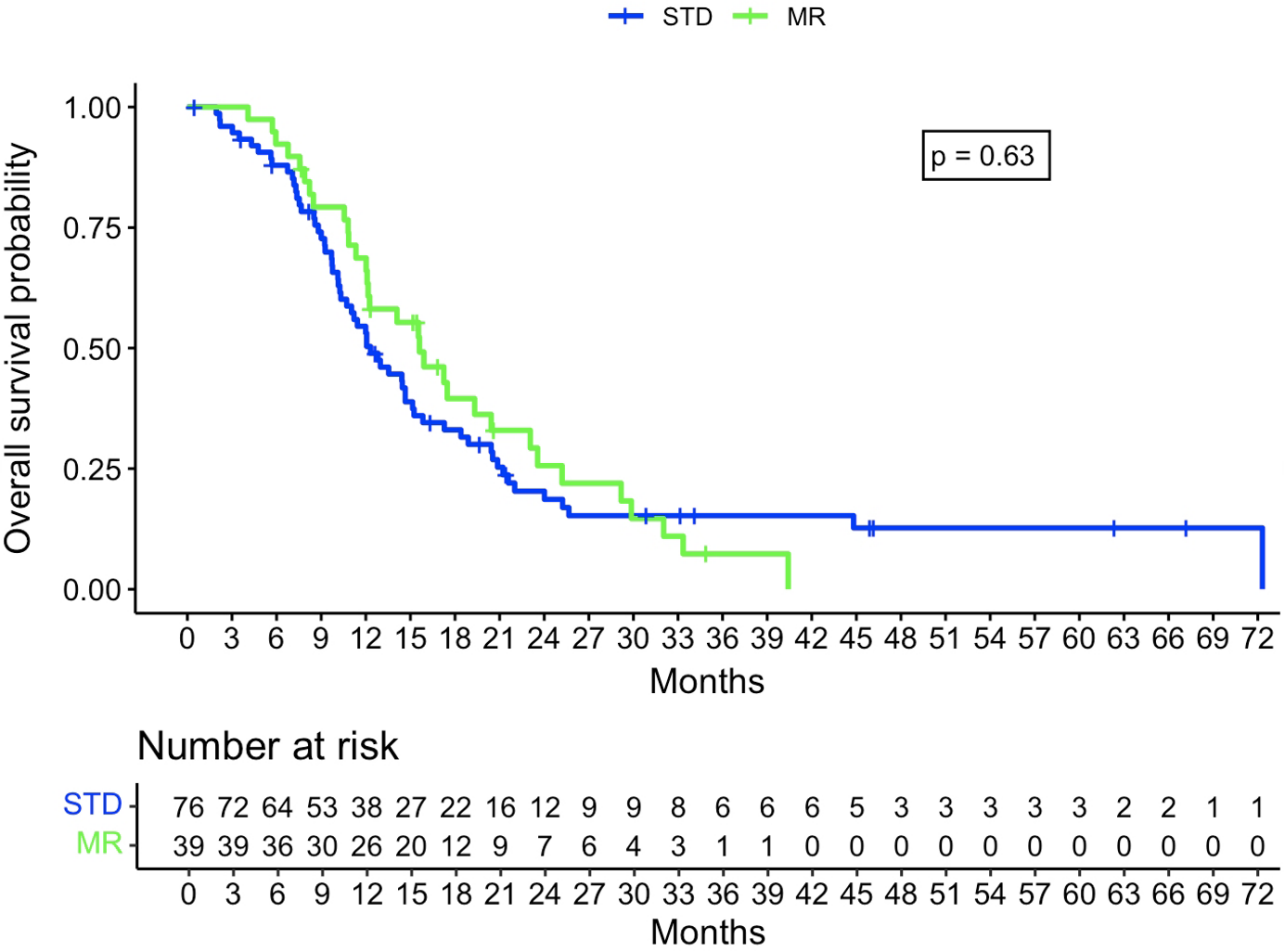
Kaplan-Meier curve regarding overall survival probability between STD and MR group. STD, Standard navigation group; MR, Mixed reality group.

**Figure 4:**
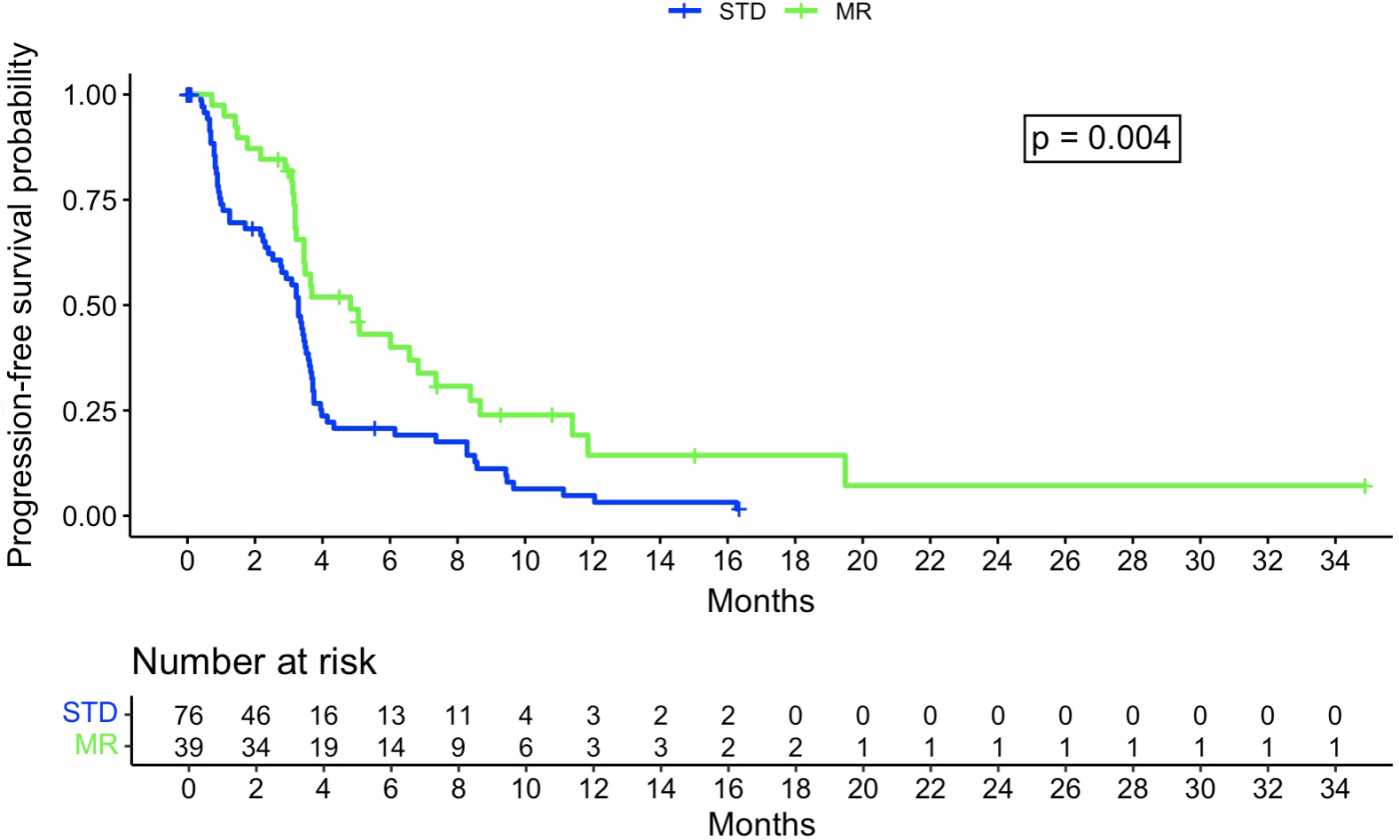
Kaplan-Meier curve regarding progression-free survival probability between STD and MR group. STD, Standard navigation group; MR, Mixed reality group.

The Kaplan-Meier analysis did not show a statistically significant difference in OS between the STD and MR groups, with, respectively, a median survival of 375 days (95% confidence interval [CI]: 314-464 days) and 475 days (95% CI: 368-717 days, p=0.63) (**Figure 3**). There was a significant difference for PFS, with a median of 100 days for the STD group (0.95CI: 84-110 days) and 147 days for the MR group (0.95CI: 105-255 days, p=0.004) (**Figure 4**).

The Cox regression model for OS had a concordance of 0.67 and relied on the following factors: use of MR, preoperative intention to remove all lesions, MGMT promoter status, and age at intervention date. This gave a hazard ratio of 0.97 for MR (p=0.9). The Cox regression model for PFS had a concordance of 0.65 and relied on the following factors: MR, preoperative intention to remove all lesions, and IONM. This gave a hazard ratio of 0.49 for MR (p=0.004).

## Discussion

This study aimed to compare the efficacy of mixed reality with standard neuronavigation applied to glioblastoma resection, considering intraoperative neuro-monitoring and 5-ALA fluorescence as confounding factors. The results demonstrate that MR significantly enhances the extent of resection and improves progression-free survival compared to standard neuronavigation.

### Mixed reality applied to glioblastoma surgery

First, a common ambiguity persists in the neurosurgical literature regarding the distinction between mixed- and augmented reality. Augmented reality (AR) refers to the adjunction of virtual images onto the real-word, whereas mixed reality involves an immersive computed-generated 3D virtual environment fitting to real anatomical structures. MR can be considered as a subtype of AR. In this way, included patients in the MR group beneficiated from MR technology as the operator was able to interact with the 3D-virtual objects, move them for a perfect congruence with the real-world anatomy, or adapt their transparency. By designing virtual objects corresponding to anatomical structures based on preoperative imaging and projecting them through the navigated surgical microscope, MR assisted the surgeon in refining their surgical approach and to improve their understanding of the patient’s specific anatomy (15). Another advantage of this technology over standard neuronavigation is its ability to maintain the neurosurgeon’s focus within the surgical field (15, 17, 19). Moreover, MR promises an enhanced precision throughout surgery compared to standard neuronavigation, owing to its possibility for real-time recalibration (17, 28). For example, navigation adjustments may become necessary during resection due to brain sagging or changes in cerebrospinal fluid levels. In our cohort, intraoperative recalibration was performed intraoperatively in an “out-to-in” manner, guided by key anatomical structures, or signature structures: virtual objects were sequentially aligned with the corresponding anatomical structures (skin, bone, cortical vessels, deep vessels or ventricles). Haemmerli et al. reported millimetric precision following recalibrations, in contrast to 3 mm achieved with standard neuronavigation (17). Moreover, in addition to offering spatial information, it also highlights connections crucial for the preservation of patients’ functionality, such as white matter tracts, based on preoperative tractography (29–32). Finally, it must be emphasized here that the use of MR during glioblastoma resection significantly increased surgical time. This is primarily due to the necessity of calibration of the surgical microscope after referencing and performing recalibrations throughout the procedure to ensure maximal precision. In summary, MR-based technologies applied to glioblastoma surgery are safe and effective, offering distinct advantages over traditional infrared standard neuronavigation.

It is important to emphasize that both STD and MR rely on preoperative imaging datasets, without the capability to adapt virtual 3D objects as the surgery progresses. Intraoperative navigated ultrasound presents an alternative that has garnered increasing interest due to its ability to provide real-time imaging updates (33). However, this technology requires the surgeon to divert their attention from the surgical field to dedicated ultrasound screens, potentially disrupting the continuity of the surgical workflow—similarly to standard neuronavigation.

### Maximal safe resection in glioblastoma surgery

In contemporary GBM treatment, achieving gross total resection (GTR) while minimizing the residual tumor volume is widely acknowledged as a critical factor influencing progression-free and overall survival (5, 6, 8, 34). Despite the infiltration of GBM into white matter, the surgical goal remains GTR, defined as the complete removal of the contrast uptake on the preoperative imaging (6, 19). Some authors have also explored supramarginal resection for glioblastomas, demonstrating improved overall survival and better seizure control compared to GTR (34–36). Preserving postoperative functional status is another crucial aspect of glioblastoma surgery (6, 8, 27, 35, 37). In our study, MR technology allowed to achieve better GTR and EOR compared to STD group. Postoperatively, 37,5% of MR group patients experienced new focal neurological deficit, similar to the STD group. Importantly, no complication was attributable to either MR or standard neuronavigation. Six weeks postoperatively, the median KPS in the MR group was 80, indicating favorable clinical outcomes. Luzzi et al. compared 54 patients undergoing supratentorial high-grade glioma resection using MR and fluorescein with 63 operated only with standard infrared neuronavigation and fluorescence (19). The authors reported GTR or near total resection above 80% with MR-assisted plus fluorescein (MR-F) surgery, significantly higher than with standard neuronavigation. Functional outcomes post MR-F surgery were also superior compared to standard neuronavigation alone.

### Confounding factors

To the best of our knowledge, Luzzi et al. were the first group analyzing the efficacy of MR (referred to as AR in their study) compared to standard neuronavigation for high-grade glioma patients (19). The authors examined high grade glioma patients undergoing surgery with the use of MR plus sodium fluorescein and compared to those operated with standard infrared neuronavigation. Sodium fluorescein is a well-established method for enhancing the resection of high grade gliomas and glioblastomas (13, 38).

In our study, all glioblastoma patients received 5-aminolevuinic acid as a standard. Similar to sodium fluorescein, 5-ALA enables fluorescence-guided surgery for high-grade glioma resection. Both fluorescence solutions are recognized as safe and effective, positively influencing OS and PFS (14). The use of intraoperative ultrasound or intraoperative MRI did not differ between the MR-group and the STD-group. Regarding IONM, the technique tended to be more frequently used in the MR-group than in the STD-group. However, IONM primarily aims to preserve neurological functions and does not directly impact the extent of resection. In summary, this study aims to isolate the impact of MR in glioblastoma surgery compared to standard navigated resection.

### Overall survival and progression free survival

Glioblastomas are known to be uncurable and typically recur, either locally or elsewhere in the central nervous system. The median survival time, even with optimal treatment, is reported to be 15-16 months (1, 15). Independent prognostic factors such as young age at diagnosis or MGMT-methylation status influence OS and PFS. In this study, MGMT-methylation status was comparable between groups. As already mentioned, intraoperative techniques, such as the use of 5-ALA or sodium fluorescein, improve OS and PFS (38, 39).

This study aimed to analyzed, as secondary outcome, the effect of MR on OS and PFS in glioblastoma patients. While MR did not significantly impact OS in this cohort, it significantly improved PFS. Our findings regarding PFS are consistent with those from Luzzi et al. (19), though there was no similar effect on OS. However, in their retrospective non-randomized study, some well-established factors predicting survival were not equally distributed across the groups, such as preoperative tumoral volume, significantly smaller in their AR group, and tumor molecular status, with wild-type IDH genotypes found in 18.5% and 33.3% of their AR and Control groups, respectively. MGMT promoter methylation was found in 74% and 57% of their groups. The authors did not perform a multiple regression analysis to correct for those confounding variables, which might have shown more mitigated results regarding AR impact on OS. Our study focused only on GBM patients (IDH wild-type genotypes), who had comparable characteristics.

It is unclear why we did not find a positive correlation with OS. We hypothesize that although RV and EOR are independent predictors of OS, the good volumetric outcomes in the STD group, though not as good as those in the MR group, may have minimized the difference in the impact of these predictive factors across the groups. Therefore, the influence of other OS predicting factors might have overshadowed the effect of improved RV or EOR, making our sample size too small to detect a significant difference.

These results emphasize the potential of new technologies applied to glioblastoma surgery aiming for maximizing safe resection of the contrast uptake on preoperative imagery to improve PFS.

### Limitations

First, our results did not to show a significant difference regarding OS, contrary to Luzzi et al. (19). As previously discussed, one explanation resides in the smaller proportion of patients included in the MR-group compared to Luzzi et al. Additionally, Luzzi et al. compared MR-assisted plus sodium fluorescein to standard infrared neuronavigation with white-light microscope. Furthermore, the relatively small sample size in our study limits the ability to observe a clear trend in overall survival (OS).

Second, this is a single-center study without randomization. A multicentric controlled randomized study should be conducted to confirm our results.

Third, augmented and mixed reality technologies are not widely available in all neurosurgical centers due to costs and accessibility. Additionally, the level of experience among neurosurgeons varies (19). Specific workshops should be organized to disseminate this intraoperative technology more broadly.

Finally, the surgical time in the MR group was 32 minutes longer than in the STD group, primarily due to the MR system setup and intraoperative recalibration processes. While this additional time may be considered a necessary investment to achieve better GTR rates with reduced RV, the development of automated intraoperative recalibration procedures could significantly minimize this extra surgical time in the future.

## Conclusions

Mixed reality applied to glioblastoma surgery demonstrates superiority over standard neuronavigation in terms of resection quality, extent of resection, and progression-free survival, without compromising patient clinical outcomes. This technology is safe and should be integrated with other intraoperative methods to guide surgeons and maximize safe resection. To address the additional time required for referencing, calibration, and intraoperative recalibrations, specific training should be provided to the neurosurgical community. This will help streamline the use of MR technology and enhance its efficacy in clinical practice.

## Data Availability

The raw data supporting the conclusions of this article will be made available by the authors, without undue reservation.
